# Searching for Semantic Knowledge: A Vector Space Semantic Analysis of the Feature Generation Task

**DOI:** 10.3389/fnhum.2019.00341

**Published:** 2019-10-04

**Authors:** Rebecca A. Cutler, Melissa C. Duff, Sean M. Polyn

**Affiliations:** ^1^Department of Psychology, Vanderbilt University, Nashville, TN, United States; ^2^Department of Hearing and Speech Sciences, Vanderbilt University Medical Center, Nashville, TN, United States

**Keywords:** hippocampus, semantic search, amnesia, relational memory, vector space model

## Abstract

A recent neuropsychological study found that amnesic patients with hippocampal damage (HP) and severe declarative memory impairment produce markedly fewer responses than healthy comparison (CO) participants in a semantic feature generation task (Klooster and Duff, 2015), consistent with the idea that hippocampal damage is associated with semantic cognitive deficits. Participants were presented with a target word and asked to produce as many features of that word as possible (e.g., for target word “book,” “read words on a page”). Here, we use the response sequences collected by Klooster and Duff (2015) to develop a vector space model of semantic search. We use this model to characterize the dynamics of semantic feature generation and consider the role of the hippocampus in this search process. Both HP and CO groups tended to initiate the search process with features close in semantic space to the target word, with a gradual decline in similarity to the target word over the first several responses. Adjacent features in the response sequence showed stronger similarity to each other than to non-adjacent features, suggesting that the search process follows a local trajectory in semantic space. Overall, HP patients generated features that were closer in semantic space to the representation of the target word, as compared to the features generated by the CO group, which ranged more widely in semantic space. These results are consistent with a model in which a compound retrieval cue (containing a representation of the target word and a representation of the previous response) is used to probe semantic memory. The model suggests that the HP group's search process is restricted from ranging as far in semantic space from the target word, relative to the CO group. These results place strong constraints on the structure of models of semantic memory search, and on the role of hippocampus in probing semantic memory.

## 1. Introduction

The most dramatic effects of hippocampal and medial temporal lobe damage are in the domain of episodic and autobiographical memory. Patients with bilateral damage to the hippocampus typically have dense anterograde amnesia, resulting in an inability to form new memories of their ongoing experience (Milner et al., 1998). This amnesic condition is consistent with the dominant view of hippocampal function: That hippocampus constructs a summary representation of the widespread cortical activity representing the details of an experienced event, and rapid synaptic plasticity binds this hippocampal representation to these widespread cortical patterns (Mishkin et al., 1983; McClelland et al., 1995; Eichenbaum, 2000). As such, hippocampus is proposed to be critically involved in binding the representations of event details to the spatiotemporal context in which they occurred, which is a defining characteristic of episodic memory (Tulving, 1972; Eichenbaum et al., 2007, 2012).

The nature of hippocampal involvement in semantic memory processes is less well settled. By one view, the hippocampus is involved in the acquisition (and possibly curation) of semantic memories through a consolidation process. Hippocampally dependent memory traces corresponding to episodic experiences are periodically reactivated, allowing cortical structures to slowly learn statistically reliable semantic characteristics of the world and the things in it (McClelland et al., 1995; Norman and O'Reilly, 2003; Eichenbaum, 2004). This view is consistent with work showing that after adult-onset hippocampal injury, the acquisition of new semantic knowledge is impaired (Gabrieli et al., 1988; Bayley and Squire, 2002; Manns et al., 2003; O'Kane et al., 2004; Sharon et al., 2011; Warren and Duff, 2014). However, it may be the case that cortical structures can form semantic memories without a functioning hippocampus. Despite dense episodic amnesia, patients with developmental hippocampal damage can still acquire new semantic knowledge (Vargha-Khadem et al., 1997). However, semantic learning in these patients seems to be slower and less flexible than in healthy individuals (Elward and Vargha-Khadem, 2018). It is possible that consolidation is better thought of as a gradual process, without a clear point at which hippocampus stops being involved (Winocur et al., 2010).

Putting aside the question of acquisition, a wide range of neuropsychological studies have shown that patients with hippocampal damage have minimal impairment in their ability to use their existing semantic knowledge. These patients perform at normal or near-normal levels on tests of their vocabulary breadth, their ability to define words and name objects, and even their ability to retrieve long-known associative pairings, such as the names of famous faces (Reed and Squire, 1998; Verfaellie et al., 2000; Schmolck et al., 2002; Westmacott and Moscovitch, 2002). In contrast, patients with damage to lateral temporal cortex, and especially anterior temporal cortex, are impaired at these semantic tasks, suggesting an anatomical dissociation of function (Irish et al., 2016). As such, the dominant view is that utilization of existing semantic knowledge does not involve hippocampus, but rather involves other cortical regions such as anterior temporal lobe (Ralph et al., 2017).

A number of recent studies have challenged this view, by demonstrating that patients with hippocampal or medial temporal lobe damage are impaired on certain tasks involving the utilization of existing semantic memory. When recounting well-known fairy tales and bible stories, these patients produce fewer details (Verfaellie et al., 2014). When producing event narratives, they use words rated lower on imageability scales (Hilverman et al., 2017), and generate fewer words in free association when cues were highly imageable and low frequency (Sheldon et al., 2013). In general, patients with medial temporal lobe damage show a retrograde impairment in retrieving information from personal semantic memory, including memories ranging back to early childhood (Grilli and Verfaellie, 2014). These findings are bolstered by periodic reports from the neuroimaging and neuropsychological literature of hippocampal involvement in semantic tasks (Henke et al., 1999; Sheldon and Moscovitch, 2012; Race et al., 2013). Furthermore, the response properties of hippocampal cells suggest that semantic information is embedded in hippocampal neural representations. For example, a substantial proportion of cells in human hippocampus show category-specific responses (Kreiman et al., 2000), and individual cells can show invariant responses to particular concepts, e.g., by responding to a particular celebrity across different images as well as to the celebrity's name presented in text (Quiroga et al., 2005; Quiroga, 2012).

A recent study by Klooster and Duff (2015) provides further evidence for hippocampal involvement in semantic memory processes. They used tasks that were developed for psycholinguistic and language-learning research, and are designed to characterize vocabulary depth and semantic richness. The Word Associates Test is an evaluative task in which a participant has to identify synonyms and collocates of a target word (collocates are words that tend to occur together in text or speech, such as *innate* and *ability*, or *maiden* and *voyage*) (Read, 1993, 1998). They also used two generative tasks. One of these was a feature generation task in which a target item was presented, and the person was asked to name as many features or characteristics of the item as they could in a 2 min interval. For example, if the target item was *book*, a participant might respond with the feature “you read words on a page.” The second was a senses task in which participants were presented with a target word and given 1 min to list senses of the word (e.g., the word *bank* can mean a financial institution, or the bank of a river). Patients were impaired on all three of these tasks relative to a set of healthy comparison participants. The most marked deficit was in the feature generation task: whereas the healthy comparison group produced upwards of 20 features on average for a given target word, the amnesic patients produced roughly half as many.

These results raise the possibility that hippocampal damage gives rise to a semantic memory deficit that is masked by patients' normal-range performance on tasks that probe semantic knowledge at a surface level. In other words, hippocampus may play an important role in semantic processing that goes beyond supporting the initial acquisition of semantic memory through replay of episodic experiences. We propose that the growing body of work establishing the role of hippocampus in relational processing may provide insight into its contribution to semantic processing. Relational processing is engaged whenever multiple arbitrary components of an experience need to be associated to one another, creating a relational representation (Rubin et al., 2014). A number of studies suggest that hippocampally dependent relational processing is engaged in a variety of cognitive domains that extend well beyond episodic memory (Cohen et al., 1997, 1999; Davachi, 2006; Olsen et al., 2012; Olson and Newcombe, 2013).

Episodic memories are inherently relational, in that an event consists of a constellation of item and contextual details that must be bound together to form a new memory trace. For similar reasons, spatial navigation involves relational processing, as the construction of representations of place and location involve processing the relations between many environmental features (Burgess et al., 2002). As such, the relational memory view of hippocampal function provides a natural explanation for why hippocampal damage is associated with both episodic and spatial memory deficits (Konkel et al., 2008; Konkel and Cohen, 2009). This account also explains behavioral deficits accompanying hippocampal damage in perceptual tasks and short-term memory tasks where the stimuli are comprised of multiple configural features (Hannula et al., 2006; Olson et al., 2006; Warren et al., 2011).

A developing branch of the relational memory literature has examined spatial reconstruction tasks, in which participants try to reconstruct a multi-item display after a short delay to evaluate spatial-relational memory. This task seems to be particularly hippocampally-dependent as participants with lesions in this area have difficulty correctly recalling the spatial relations of studied items (Smith and Milner, 1981; Jeneson et al., 2010; Watson et al., 2013; Horecka et al., 2018). A subset of multi-item spatial encoding tasks have found evidence for a role of the hippocampus in actively guiding visual search, with hippocampal activation corresponding to enhanced subsequent memory (Voss et al., 2011a,b; Lucas et al., 2018). We consider the idea that internal semantic feature search may in some ways parallel navigation and visuospatial exploration, given that the hippocampus seems to facilitate information-gathering and sampling in both processes. We propose that semantic deficits due to hippocampal damage are related to previously observed relational processing deficits.

### 1.1. A Computational Analysis of the Feature Generation Task

In the current study, we examine the data originally collected by Klooster and Duff (2015) to test whether participants' impaired performance on the feature generation task can be understood in terms of a relational semantic deficit. To do this, we use a computational model of semantic representational structure to characterize the memory search processes engaged by the task. This allows us to examine the semantic relations between generated features and the target word, and the relations of the set of generated features to one another. We find substantial differences in the nature of semantic search between the two groups, which we interpret in terms of current theories of semantic and episodic memory search. While Klooster and Duff (2015) also characterized semantic deficits in two other tasks, feature generation performance was the most amenable to semantic analysis: its generative nature allowed us to examine the dynamics of search, and participants overall produced about five times as many responses in this task relative to the senses task.

A number of algorithms have been developed to construct semantic representational codes from either large text corpuses (Lund and Burgess, 1996; Landauer and Dumais, 1997; Jones and Mewhort, 2007) or from behavioral responses in a free association task (Steyvers et al., 2004). These are often referred to as vector space models of semantics, as each representational code in the system is a vector of numbers. While the numerical features that comprise the representations in these vector space models are rarely directly interpretable, they do provide a reference point for each word, such that words with similar features are situated near to one another in the vector space. This computational approach allows us to consider a sequence of responses in the feature generation task as a trajectory through an abstract semantic representational space. This trajectory can be characterized in terms of the semantic distance between the target word and the individual features generated by participants, and the distance of the generated features to one another.

Hills et al. (2012) used a similar approach to characterize performance on a semantic fluency task, in which participants are asked to provide examples from the semantic category “animal.” In their model, the vector representation of the most recent response was used as a retrieval cue to determine the next response. The likelihood of recalling a particular word in a search of the category semantic space was proportional to its representational similarity to the most recent response. This framework naturally explains the semantic clustering seen in semantic fluency tasks: The initial response tends to be a highly frequent exemplar of the category (Henley, 1969; Newcombe, 1969), and the continual updating of the retrieval cue causes contiguous responses to be semantically similar to one another (Bousfield and Sedgewick, 1944; Federmeier et al., 2002; Voorspoels et al., 2014).

Our proposed model is similar to the Hills et al. model in that feature responses are based on a blended representation of target word and previous recall information. Critically, all of the HP patients in the (Klooster and Duff, 2015) study were impaired in feature generation. However, as a group they did not show a deficit in a measure of semantic fluency (the Controlled Oral Word Association test), although there seems to be more variability in their performance at the individual level. It is therefore important to understand how semantic feature generation differs from semantic fluency, and how the task demands might reveal the nature of semantic deficits in hippocampal amnesia. In both cases, the participant's knowledge is probed in a constrained way. With semantic fluency, responses are constrained to come from a particular taxonomic category (Gruenewald and Lockhead, 1980). In semantic feature generation, responses are constrained to be in reference to a target word, and are meant to describe properties or characteristics of the referent item. However, these tasks seem to require access to different kinds of conceptual representations. Firstly, the feature generation task cues semantic search with a more specific target than semantic fluency (e.g., “dolphin” vs. “animal,” respectively). Secondly, the task demands of feature generation requires retrieval of richer multi-word conceptual representations, whereas semantic fluency requires participants to name exemplars. Lastly, in semantic fluency each response is related to the others by the shared features that comprise category membership. In comparison, in semantic feature generation the adjacently retrieved features are potentially semantically unrelated outside the context of the target word (e.g., “gray in color” and “intelligent animal” for “dolphin”). This type of feature generation seems to require relational memory to access semantically disparate concepts that are related only given the context of the target word. Our semantic analyses suggest that this task distinction is important and can potentially unearth semantic memory deficits that are otherwise masked in surface-level tasks. In the discussion we consider the critical role of the hippocampus in relational memory, and the key differences between the tasks mentioned above and semantic tasks which are not associated with an impairment in patients with hippocampal amnesia. We will return to the question of how these results inform our understanding of hippocampal engagement in semantic memory search.

## 2. Methods

### 2.1. Participants

Participants were five patients with bilateral hippocampal damage (HP) exhibiting declarative memory impairments. Fifteen healthy participants (CO) were matched to the patient group on sex, age, and education (three matched participants to each patient). Each patient with hippocampal damage had stable, non-progressive lesions. The etiology of three patients was anoxia/hypoxia—resulting in bilateral hippocampal damage. Two patients had herpes simplex encephalitis, resulting in broader bilateral medial temporal lobe damage, including hippocampus, amygdala, and surrounding cortices. For more details see Klooster and Duff (2015).

### 2.2. Experimental Procedure

In the feature generation task, participants were presented with a target word (e.g., “bed”) and given 2 min to verbally list as many features of that word as possible. Thirty-five target words were sampled from established feature production norms (McRae et al., 2005). Instructions and examples were given to the participant at the start of the task, and were left in front of the participants and repeated by the experimenter regularly. On each trial, the experimenter read the target word aloud, and prompted the participant to begin to report features. If, during this recall period, the participant stopped responding, the target word was repeated by the experimenter, and the participant was encouraged to keep trying to generate features. Responses were video recorded for later transcription and analysis.

### 2.3. Preprocessing Response Sequences

Each participant's verbal responses were transcribed and two judges coded these responses into a sequence of features. See Klooster and Duff (2015) for details regarding this coding procedure. Table 1 provides representative examples of features. For the current study, we developed a coding scheme to include all content words and exclude function words from the semantic analysis. Excluded grammatical groups were: personal pronouns, possessive pronouns, auxiliary verbs (be, do, and have), coordinating conjunctions and articles.

**Table 1:** Examples of features generated by healthy comparison participants (CO) and patients with hippocampal damage (HP) for the target words “book” and “grapefruit.”

**Group**	**Target word**	**Feature (word similarity below)**				**Cosine**
CO	Book	Can be **bound** in either **leather** or **paper**				0.2911
			0.2328	0.1809	0.4542	
HP	Book	**Something** you **read**				0.4686
		0.3614	0.5758			
CO	Grapefruit	**Often**	**produced** in **Florida**			0.0531
		–0.0674	0.0560	0.1708		
HP	Grapefruit	**Skin**	**like**	**oranges**		0.2829
		0.1805	0.0666	0.6017		

*Bolded words in each feature indicate which words were included in the analysis after the exclusion of non-content words. Underneath each bolded word is the cosine similarity score between that word and the target word. These values were averaged to create the overall cosine similarity score for the feature, which is given in the rightmost column*.

### 2.4. Semantic Vector Space Models

In this study we used semantic representations constructed with the Global Vectors (GloVe) algorithm (Pennington et al., 2014), which has excellent coverage of the English language due to the large text corpus used to construct the vector representations. GloVe follows in a long tradition of computational models attempting to quantify the meaning of words by assigning each word a point in a high-dimensional vector space, often containing up to 300 dimensions (Deerwester et al., 1990; Lund and Burgess, 1996; Landauer and Dumais, 1997; Steyvers et al., 2004). These techniques tend to use linear algebraic algorithms (such as singular value decomposition) to construct vector representations given statistics characterizing the co-occurrence of words in a large text corpus.

These semantic vector space models formalize longstanding ideas from linguistics and philosophy regarding how best to characterize the meaning of words. In the linguistics literature, the *Distributional Hypothesis* refers to the notion that words that co-occur across similar contexts tend to have similar or related meanings (Harris, 1954). Linguist J. R. Firth famously summarized the context-dependent nature of meaning with the phrase “You shall know a word by the company it keeps” (Firth, 1957, p. 11). The assignment of vector representations to words and phrases resonates with ideas developed by Wittgenstein (1953), whereby words can be loosely grouped by a combination of shared features. Given these vector representations, the semantic similarity of two words can be quantified using standard distance measures such as Euclidean distance or the cosine angle between two vectors (Kwantes, 2005). In the current work we used the GloVe model to construct a single representation for multi-word features by taking the average of the semantic similarity of each of the feature words to the target word. For feature-to-feature analysis we calculated the pairwise similarity between each word in the two features; the average of these similarity scores was used to represent the similarity of the features to one another.

### 2.5. Bayesian Analysis of Feature Responses

We implemented all Bayesian analyses in R. Initial analysis using a frequentist framework indicated that residuals were not normally distributed, motivating the use of a Bayesian framework. Further, the nature of cosine values is such that they are log-normally distributed, and a Bayesian framework gives us more flexibility to estimate this. To examine group differences in cosine similarity scores we fit a Bayesian linear mixed effects model using the Stan and brms packages in R (Bürkner, 2017; Stan Development Team, 2017). The binary group predictor (CO vs. HP) was modeled as a fixed intercept and slope. Subject (*s*) and target word (*w*) predictors were modeled as random effects with varying intercepts and normally distributed priors: *s* ~ *Normal*(0, σ*s*), *w* ~ *Normal*(0, σ*w*). Prior distributions on the variance parameters were uniform: σ*e*, σ*s*, σ*w* ~ *Uniform*(0, ∞). We estimated the response distribution of cosine similarity scores as log-normal, as preliminary examination of the data showed that this distribution was better described with a log-normal distribution as compared to a normal distribution. We estimated model parameters using Markov-Chain Monte-Carlo (MCMC) methods, using the No U-turn Sampler (NUTS) provided with Stan. For all Stan-based model fits, we ran 4 chains each with 4,000 iterations to ensure chains effectively converged. Chain convergence was confirmed by the $\hat{r}$ statistic which in all cases approached 1 (indicating maximal convergence).

A second Bayesian linear mixed effects model was designed to characterize the cosine similarity of feature responses to one another (within a given response sequence). Similarity was calculated between features with lag one to four. Lag is defined as the positional difference in the response sequence, with adjacent features assigned a lag of one, features separated by one intervening feature assigned a lag of two, and so forth. Group, subject, and word predictors were modeled as described above, except that the response distribution of cosine similarity scores was modeled as normally distributed. Prior distributions on the variance parameters, MCMC details, and model comparison details were the same as above.

In order to examine changes in cosine similarity across the response sequence, and changes as a function of lag within a given response sequence, we created a set of Bayesian multilevel models. The data was best modeled by power functions, which take the form *f*(*x*) = *a*(*x*^*b*^), where *a* is a scaling factor, and *x* is a variable base raised to a constant power, *b*. The *b* coefficient represents the growth or decay in cosine similarity scores as a function of *x*, which represents either response position or positional distance between generated features in different analyses. In our two-level hierarchical models, we estimate the group and subject-level effects of feature responses. At the group level, we estimated the *a* parameter for both groups with the prior μ ~ *Normal*(0.2, 0.5), σ ~ *Cauchy*(0, 5), and the *b* parameter with the prior μ ~ *Normal*(0, 0.5), σ ~ *Cauchy*(0, 5). We estimated model parameters using MCMC in Stan as described above. All chains converged effectively. As above, these models were compared to a null model without a group-level predictor.

## 3. Results

### 3.1. Group-Level Shift in Target-Feature Relatedness

**Figure 1:**
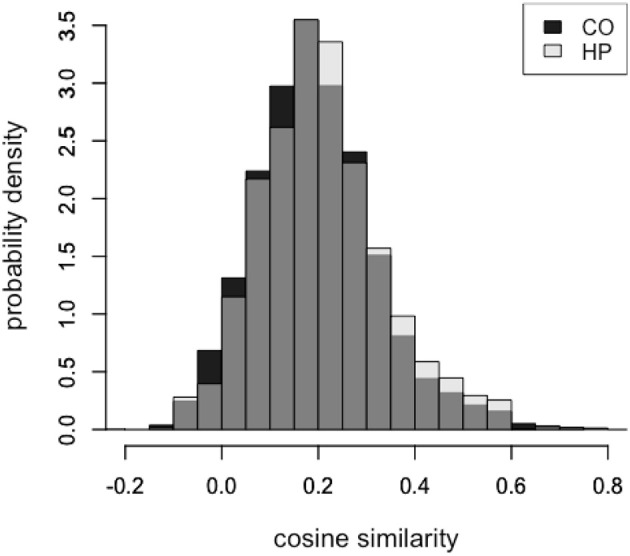
Feature responses in the hippocampal patient group were more highly semantically related than matched comparison participants, across all target words. Normalized distributions of overall cosine similarity in the HP participants (light) and CO participants (dark). Gray indicates regions of overlapping distributions.

Overall, the feature responses made by patients with hippocampal damage tended to be closer in semantic space to the target word (HP: μ = 0.21, *SD* = 0.13) when compared to healthy comparison participants (CO: μ = 0.19, *SD* = 0.13). This positive shift in the cosine distribution of HP responses can be seen in Figure 1. We used a Bayesian mixed effects regression framework to investigate the effect of group (HP vs. CO) on cosine similarity of feature response to target word. The model had a fixed effect of group and accounted for variance associated with individual subjects and target word stimuli. The posterior distribution of the MCMC chains for the group coefficient did not include zero (μ = 0.02, *SD* = 0.003, 95% CI = [0.002, 0.033]), consistent with a substantial and reliable group difference in cosine similarity. We considered the possibility that the increased cosine similarity of features to target word was driven by an individual patient, therefore we carried out a “leave-one-out” by patient analysis. We iteratively ran the model described above five times, each time excluding one patient's data. For each iteration, the resultant posterior distribution of the group parameter did not include zero, suggesting that no single patient was skewing the group result.

### 3.2. Feature-to-Target Relatedness Across Response Positions

**Figure 2:**
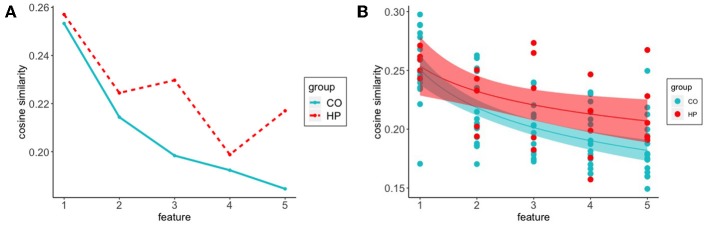
Cosine similarity of target word to feature responses decreased across the first five responses in both groups. CO participants show a steeper decline compared to a more gradual outward trajectory from target word space in HP participants **(A)** Average cosine similarity of features to target word for responses 1:5 for CO (-) and HP (- -) groups **(B)** Power function fit to HP (*a* = 0.2536, *b* = –0.1243) and CO (*a* = 0.2510, *b* = –0.2018) initial five responses. Shadows represent 95% confidence intervals.

Firstly, we were interested in how participants initiate the search for features of a given concept in semantic space. We examined the cosine similarity between the target word and the first five feature responses. Participants in the HP group generally make fewer responses than the CO participants, but routinely make more than 5 responses. As such, restriction to the first 5 responses puts the two groups on relatively even footing in terms of the number of responses in each response position bin. In both HP and CO groups we found that the cosine similarity to the target word decreased across the first five responses (see Figure 2). While the first feature response of both groups was a similar distance from the target word, a group difference emerges over the course of the first five responses, with the CO responses ranging farther in semantic space on average relative to the HP responses.

To characterize these observed trends, we constructed a Bayesian hierarchical model, fitting power function curves to this sequence of response positions. Best-fit curves are presented in Figure 2B. The best-fit curves for the two groups have similar starting points (as reflected by the best-fitting *a* parameters, HP: 0.2536, CO: 0.2510), but the patient group shows a slower rate of decay in semantic similarity to the target word as the search progresses (as reflected by the best-fitting *b* parameters, HP: –0.1243, CO: –0.2018).

In order to characterize the statistical reliability of this difference in decay rates, we examined the MCMC-derived posterior distributions for each of the power function curve parameters. Intuitively, these posterior distributions contain the set of plausible parameter values for each of the groups. As we are interested in determining whether the shift in parameter values is reliable, we constructed a *difference distribution* for each of the parameters: Each sample in the posterior distribution specifies four numbers, the mean *a* parameter for the HP and CO groups (ā_*HP*_, ā_*CO*_), and the mean *b* parameter for the HP and CO groups (${\bar{b}}_{HP},{\bar{b}}_{CO})$. The difference distributions were constructed by calculating ā_*HP*_−ā_*CO*_ and ${\bar{b}}_{HP}-{\bar{b}}_{CO}$ for each sample in the posterior distribution.

For the *a* parameter, the mean of the difference distribution was near-zero (0.0027), with points tending to be evenly distributed around zero (in 57% of posterior samples ā_*HP*_>ā_*CO*_). This suggests that both groups initiate semantic search in a similar way. For the *b* parameter, the mean of the difference distribution was more substantially positive (0.0762, consistent with a shallower decay for HP), with 83% of the difference distribution falling above zero. In other words, the semantic relatedness of the generated features to the target word decayed more slowly for the HP group, consistent with the idea that the CO group is able to range further from the target word in semantic space. The group difference in the *b* parameter is consistent with the group difference established in the first analysis, but is less reliable statistically. This is likely due in part to the restriction of this analysis to the first five response positions, and also to the presence of fairly substantial individual differences, as can be seen in Figure 2B.

Considering the first two analyses together, it seems reasonable to infer that the group-level difference characterized in the first analysis is not present in the initial responses. This is consistent with a model in which the difference in semantic relatedness emerges over the course of the response sequence. A follow-up analysis showed that the mean target-to-feature semantic relatedness for the later response positions excluded from this analysis (through to the termination of the response sequences) is similar to the asymptotic values approached by the two power curves estimated here. These results are generally consistent with a model in which the hippocampus facilitates the retrieval of semantically distant features of the target word. We return to this point in the discussion.

### 3.3. Feature-to-Feature Semantic Relatedness

**Figure 3:**
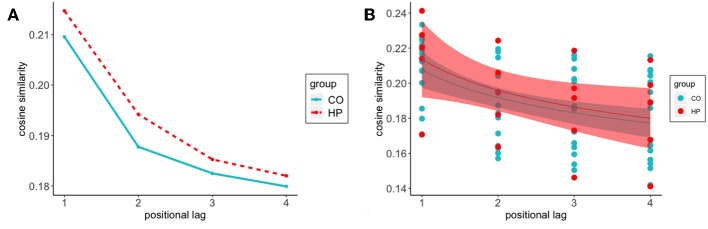
Feature-to-feature cosine similarity decreases as the positional distance between them increases in the response sequence to a given target word. In general, HP patient's responses are more semantically related to one another as compared to CO participants **(A)** Average cosine similarity between feature positions at lag 1:4 for HP (-) and CO (- -) **(B)** Power function fit to HP (*a* = 0.2131, *b* = –0.1256) and CO (*a* = 0.2077, *b* = –0.1184) average cosine similarity of response positions from lag 1:4. Shadows represent 95% confidence intervals.

The previous analyses examined the semantic similarity of the words comprising each feature to the target word specific to that trial. In order to better characterize the dynamic nature of semantic memory search, we calculated the semantic relatedness of the reported features to one another, without regard to the semantic identity of the target word. This allowed us to examine how feature-to-feature similarity changed as a function of the relative position of the two features in the response sequence. Figure 3 shows that as the positional lag between two responses on a given trial increases, there is a substantial decline in cosine similarity. In other words, as two responses become farther apart in the response sequence, they become less semantically related to one another.

We first used a Bayesian linear mixed effects model to estimate average feature-to-feature semantic relatedness, without considering transition lag. As before, the model had a fixed effect of group and accounted for variance associated with individual subjects and target words. The posterior distribution of the group coefficient was centered around zero (μ = 0.0068, *SD* = 0.0119), consistent with the idea that there is no HP vs. CO group difference in feature-to-feature similarity.

Once more, we constructed a Bayesian hierarchical model, fitting power functions to these curves to determine whether a group difference exists in the non-linear shift of semantic relatedness as feature responses become separated in the response sequence. Best-fit curves are presented in Figure 3B, with parameter estimates for each group in the caption. The best-fit curves for the two groups have similar starting points (the *a* parameter, HP: 0.2131, CO: 0.2077), and similar decay in semantic similarity as the positional distance between responses increases (the *b* parameter, HP: –0.1256, CO: –0.1184). As above, we calculated difference distributions, ā_*HP*_−ā_*CO*_ and ${\bar{b}}_{HP}-{\bar{b}}_{CO}$, for each sample in the posterior distribution. For the *a* parameter, the mean of the difference distribution was near-zero (0.0059), with 65% of the samples in the CO group less than the HP group. For the *b* parameter, the mean of the ${\bar{b}}_{HP}-{\bar{b}}_{CO}$ distribution was also near zero (0.0079) with 58% of the HP samples falling below the CO samples. This analysis suggests that the process governing transitions between generated features behaves similarly for the two groups.

## 4. Discussion

We used a vector space model of semantic meaning to investigate differences in how patients with hippocampal damage (HP) and healthy demographically matched comparison participants (CO) performed on a feature generation task. Our results are consistent with the idea that the hippocampus is important for relational semantic memory. We constructed semantic representations of the multi-word features produced by both groups, and examined the representational similarity of these features to the target word representation, and to other features reported in the same trial. We found that there was a group difference in the overall similarity of features to the target word, such that HP patients tended to generate features that were more semantically related to the target word, relative to the CO group. Furthermore, while both groups initiated search at a similar semantic distance from the target word, a difference emerged across the subsequent responses. Feature-to-target semantic similarity generally declined across responses within a trial for both groups, but the decline was reliably steeper for the CO group, consistent with their tendency to produce responses that were on average more distant from the target word in semantic space. We also found evidence for local transitions in a structured semantic space. On a given trial, adjacent features in the response sequence were most similar to one another, with similarity declining steadily as the lag between responses increased. In the following sections we discuss the motivation for using semantic vector representations to model this task, and how they inform our understanding of a relational semantic memory deficit with hippocampal damage.

### 4.1. Search Process in Semantic and Episodic Memory

We consider how semantic vector space representations could work with mechanisms commonly used to model search processes in episodic and semantic memory. In order to develop a model of the semantic feature generation task, we begin by comparing it to other memory search tasks that have been modeled computationally. In many theories, memory search is guided by the construction and utilization of a retrieval cue: a mental representation that targets and reactivates task-relevant memories. For example, in the semantic fluency model developed by Hills et al. (2012), a retrieval cue containing the most recently reported response was used to target local conceptual representations from the category “animal.” The representational similarity between the retrieval cue and each of the not-yet-recalled animals was used to simulate a decision competition in which the likelihood of a given animal winning the competition was proportional to its semantic similarity to the retrieval cue. The continual updating of the retrieval cue causes contiguous responses to be semantically similar to one another. Smith et al. (2013) used a semantic vector space model to examine the search process in a Remote Associates Test in which the participant must produce a target word that is semantically related to three presented cue words. Participants were encouraged to vocalize guesses as they attempted to determine the target word, and their model suggested a similar semantic dependence of a given response on the previous responses in the sequence. It is worth noting that hippocampal patients in the Klooster and Duff (2015) study were impaired on a similar Word Associates Test, in which remote semantic associates to a target word had to be identified.

In retrieved-context models of free recall, a retrieval cue comprised of context information is used to target episodic representations of words from a recently studied list (Howard and Kahana, 2002a; Sederberg et al., 2008). In many experiments, the temporal structure of the studied items dominates clustering during the recall period: items studied in nearby list positions tend to be recalled in adjacent output positions. In these models, recalling an item reinstates the context associated with that item at encoding which increases support for its neighbors at retrieval (Kahana, 1996; Kahana et al., 2008; Healey et al., 2019). There is a simultaneous influence of semantic relatedness on the order of recall responses (Howard and Kahana, 2002b; Polyn et al., 2009). As in the semantic fluency task, semantically related study items tend to be produced as contiguous responses in the recall sequence (Romney et al., 1993; Polyn et al., 2011). In a number of free recall models, these semantic organization effects arise from the dynamics of an ever-changing retrieval cue which integrates the representation of the just recalled item (Sirotin et al., 2005; Polyn et al., 2009; Socher et al., 2009; Morton and Polyn, 2016). Here we consider how similar mechanisms could be used to develop a model of the semantic feature generation task.

### 4.2. Toward a Mechanistic Model of the Feature Generation Task

The current results provide constraints that can be taken into account in future modeling work. With regard to the functioning of the healthy cognitive system, we envision an executive system guiding task performance through the construction of a retrieval cue that probes a semantic memory space. This semantic memory space is populated with representations of known objects as well as representations of their features and characteristics. The retrieval cue activates a particular location in this semantic space, which activates nearby conceptual representations in proportion to their proximity to the activated location. This proximity-based activation is similar to the dynamics of a spreading activation model (Collins and Loftus, 1975; Anderson, 1983). These representations then compete to be retrieved, with their relative activity determining the support for each representation. The cosine similarity scores used in our analyses reflect the proximity of these representations to one another, and as such, can be thought of as approximating the level of support for each representation in this retrieval competition. The winning representation is fully activated, allowing that feature to be verbally reported. The retrieved feature representation can then be used to modify the retrieval cue, and semantic search continues.

**Figure 4:**
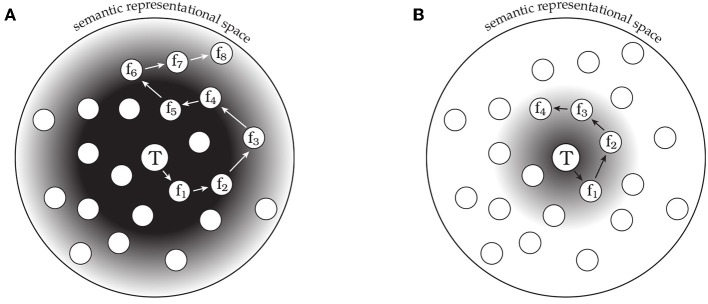
A schematic model of semantic memory search differences in healthy comparison participants (CO, **A**) and patients with hippocampal damage (HP, **B**). The central circle labeled T corresponds to the conceptual representation of the target word, and the surrounding circles correspond to potentially reportable features. The gradient represents the strength of support for features based on the target concept. Arrows indicate the sequence of feature responses (f_*n*_) made by the hypothetical participant. The tendency to make transitions to nearby features suggests a search process that is influenced by the most recent response. **(A)** CO participants make a longer sequence of responses that ranges farther in semantic space. **(B)** HP participants produce a shorter response sequence that does not range as far from the target concept.

The observed behavioral phenomena are consistent with this model. We propose that semantic space is probed and guided by a compound retrieval cue, containing a representation of the target word as well as a representation of the most recent feature response. The first features retrieved tend to be close in semantic space to the target word, suggesting that the initial search is guided by a retrieval cue that simply contains a representation of the target word. Subsequent responses range further from the target word in the semantic space, and neighboring responses tend to be more similar to one another than to other responses. One way for the system to support retrieval of more distant features in the semantic space is to integrate information related to already retrieved features into the retrieval cue itself, creating a compound cue of target and recent feature information. This compound cue would allow the system to target more distant parts of the semantic space, as features proximal to the already retrieved features would now receive additional support in the retrieval competition. By retaining target word information in the retrieval cue, the system can ensure that retrieved features remain relevant to the current target word. However, as the number of feature responses increases, the target word representation may become progressively less influential in the retrieval cue, allowing the system to range further from its point of initiation (as shown in Figure 4).

These results also provide constraints regarding the specific contribution of the hippocampus to semantic memory search, although there remain a number of open questions that we discuss in the following sections. Specifically, with hippocampal damage, feature responses have a restricted range in semantic space. However, at the same time the semantic relatedness of successively reported features to one another is unaffected. This raises the possibility that the executive machinery guiding search is unaffected, as it is still able to incorporate information about the previous response into the retrieval cue guiding search. Furthermore, patients are able to reliably stay “on task,” in that they consistently generate valid features of the target word. Indeed, Klooster and Duff (2015) found no significant group difference in the number of unrelated responses (*p* > 0.27) or the number of factually incorrect responses (*p* > 0.62) produced between the CO and HP groups. The deficit seems more specific to the patients' ability to access distant semantic features of the target word.

### 4.3. Hippocampal Damage and Semantic Memory

As reviewed in the introduction, the hippocampus has been clearly implicated in both relational processing and episodic memory. However, its role in semantic memory is less well characterized. We propose that people with hippocampal damage have difficulty using semantic knowledge in a flexible, relational manner. As mentioned above, neuropsychological studies have found that patients show minimal impairment in basic tasks probing semantic knowledge, but it is possible that these tasks mask a more subtle deficit in relational processing.

A number of studies indicate that the hippocampus contributes to successful relational memory – that is, the formation of long-term memories comprised of multiple elements bound together (Cohen et al., 1999; Konkel and Cohen, 2009). As we discuss below, relational processes can be independent of long-term memory and can refer to any cognitive mechanism involving relational representations. In the feature generation task the HP group shows an impairment in retrieving rich semantic representations: fewer features are produced, and the produced features do not range as far in semantic space as those produced by the CO group. We discuss possible explanations for this observed deficit. First, we consider that the relational search process, facilitated by a compound retrieval cue, is impaired. Second, we consider whether the deficit could arise directly from an impaired ability to retrieve episodic memories. Third, we explore the possibility that the HP group impairment is due to a general degradation of the semantic space used to represent features.

#### 4.3.1. Relational Binding and the Hippocampus

The relational-binding theory of memory posits that the hippocampus plays a critical role in assembling and relating the disparate details of an experience to form a coherent, holistic representation (Cohen and Eichenbaum, 1993; Ryan et al., 2000; Davachi and Wagner, 2002; Barense et al., 2007; Staresina and Davachi, 2009; Olson and Newcombe, 2013). As such, hippocampal damage affects performance on a variety of tasks outside of the domain of episodic memory. Here, we consider the relevance of this theory to the semantic deficit observed in the feature generation task. By this theory, semantic memories may be generally intact in HP patients. The impairment would arise from an inability to hold multiple or diverse semantic features in mind simultaneously to probe semantic memory effectively.

A number of studies have shown that patients with hippocampal damage have impaired memory for configural information at very short delays (Hannula et al., 2006; Warren et al., 2015), and even when all relevant information remains onscreen (Warren et al., 2011, 2012). Warren et al. (2011) reported an impairment in amnesic patients performing visual search for a target among complex stimuli which resemble the target to varying degrees. In order to perform this visual search task, one likely has to construct and maintain a complex internal representation of the target stimulus. This internal representation could then be used to determine whether a given lure stimulus matches the target. They found that while comparison participants fixated on the target less often as the trials went on, patients fixated on it at a constant rate across trials, suggestive of hippocampal involvement in maintaining the complex representation of the target item. More recently, Lucas et al. (2018) found that patients with hippocampal amnesia were more likely to engage in random, less structured saccade patterns when studying a spatial array. This randomness was predictive of less accurate spatial reconstruction, consistent with the idea that the hippocampus helps construct and maintain a configural representation of the spatial environment.

Further evidence for a hippocampal role in exploratory viewing comes from neuroimaging studies looking at the fMRI BOLD response in the hippocampus as participants controlled which item they studied in a spatial array (Voss et al., 2011a,b). A key finding was that “spontaneously revisiting” an item (i.e., looking backward at a recently viewed item) produced a subsequent memory benefit for that item and was associated with increased hippocampal connectivity. Interestingly, patients with hippocampal amnesia rarely engaged in this revisiting behavior, suggesting a causal role of the hippocampus in strategic learning of the spatial array (Voss et al., 2017).

The ability to construct or maintain a complex configural representation may be generally important for tasks involving cognitive search (Pachur et al., 2012). A recent paper by Solomon et al. (2019) considers this possibility in their examination of intracranial electroencephalographic activity during episodic memory search. They found correlations between hippocampal theta oscillations, and distances between studied items in both temporal (list position) and semantic (word meaning) spaces. As hippocampal activity has already been implicated in coding of spatial environments (O'keefe and Nadel, 1978), these results raise the possibility that hippocampus has a domain-general role in the formation, maintenance, and utilization of cognitive maps of any kind of information.

The visual search results described above are also consistent with the possibility that hippocampus supports search by allowing one to periodically “refresh” a target representation through episodic retrieval. By this account, the deficit in the feature generation task could arise from a difficulty in holding the target word in mind; if the representation of this word is disrupted, an HP patient would be unable to refresh it and continue the search. However, Klooster and Duff (2015) also found that patients were impaired in another semantic task, the Word Associates Test (WAT). In the WAT, all relevant materials are presented simultaneously and remain in view throughout the trial, obviating the need to rely on, or refresh, a representation held in memory. As such, we propose that the critical commonality between the semantic feature generation task and the WAT is the need to hold multiple disparate semantic features in mind simultaneously as part of a retrieval cue, in order to more effectively probe semantic memory. If the HP patient group is impaired in their ability to construct and maintain this retrieval cue, their ability to probe semantic space will be limited, regardless of whether the semantic space itself is degraded.

Relevant to this point, two other impairments related to hippocampal damage bear mentioning. First, individuals with hippocampal amnesia have difficulty forming a coherent mental image of a familiar scene during an imagination task. Fragmented images can be generated, but patients are impaired in relating these to one another to create a holistic representation (Hassabis et al., 2007). Second, individuals with hippocampal amnesia are impaired at constructing semantic narratives that are not autobiographically relevant (e.g., a fairy tale), producing fragmented narratives without clear temporal structure (Rosenbaum et al., 2009). These findings are consistent with a framework in which cognitive deficits in patients with hippocampal damage are not necessarily due to a deficit in the ability to retrieve experiences from memory *per se*, and more so due to a difficulty in assembling and relating disparate details to form a coherent, holistic representation (Kwan et al., 2013)

#### 4.3.2. Alternative Possibilities Regarding the Feature Generation Deficit

Two hypotheses regarding the functional consequences of hippocampal damage are worth considering. First, the possibility that the observed semantic deficits arise from an inability to retrieve autobiographical episodic memories during task performance (Ryan et al., 2008; Greenberg et al., 2009; Greenberg and Verfaellie, 2010), and second, the possibility that semantic knowledge is generally degraded by the absence of a hippocampally mediated consolidation process.

Under the first hypothesis, participants would draw upon autobiographical memories of interacting with a target item in order to generate semantic features. Indeed, in the data collected by Klooster and Duff (2015) participants sometimes retrieve episodic memories in order to generate semantic features (e.g., for the target word “key,” “*I've got a padlock that your key sticks in and it actually screws the padlock shut”*). However, Klooster and Duff (2015) found no reliable differences in the frequency with which each group used personal anecdotes in their responses. Furthermore, the same amnesic patients showed semantic impairments in the Word Associates Test (WAT). As described above, the WAT tests the depth of one's vocabulary knowledge, asking participants to decide which of several simultaneously presented words are related to a target word (either by meaning or collocation). It is unclear how drawing upon one's autobiographical experience would help in this task. Furthermore, work characterizing a class of memories termed personal semantics suggests that in some cases the distinction between semantic and episodic memories may not be clear cut (Renoult et al., 2012). Some types of personal semantic memories are thought to be hippocampally dependent (e.g., memories for repeated or regularly recurring events), supporting the idea that there is not necessarily a rigid dichotomy between the episodic and semantic memory systems.

Under the second hypothesis, the periodic replay of episodic memories interleaves reactivation of older semantic memories and newly acquired information, limiting interference between older and newer memories, and generally curating one's semantic memories. Without a hippocampus, it is possible that the semantic knowledge store is not sufficiently maintained, causing the representations to degrade over time (McClelland et al., 1995; O'Reilly and Rudy, 2001; O'Reilly and Norman, 2002). This could make it more difficult to retrieve information from semantic memory. In terms of the vector space model, degradation of the semantic representations (e.g., by adding noise to them) would tend to make related concepts become more distant from one another. This could explain why patients with hippocampal damage retrieve fewer features and preferentially retrieve features that are close in semantic space, as the more distant concepts may have become so distant as to be inaccessible. We believe this possibility deserves further consideration. The development of a more refined computational model of semantic search may prove informative. Such a model could examine whether the data are more consistent with a model in which hippocampus supports the search process itself (by allowing the discovery of more distant semantic relations) as opposed to a model in which hippocampus is not involved in the search process, but curates the knowledge being searched over.

## 5. Conclusions

Vector space models of semantic representational structure are valuable tools for the characterization of performance on semantic memory tests. Here, we showed that patients with hippocampal amnesia have difficulty generating features that are semantically distant from a target word. However, the semantic relatedness of produced features to one another was unaffected. These results are broadly consistent with relational theories, in which hippocampus facilitates exploration of any cognitive representational space. We hope that these results will prove informative for future efforts to develop mechanistically explicit models of semantic memory search.

## Data Availability Statement

The raw data supporting the conclusions of this manuscript will be made available by the authors, without undue reservation, to any qualified researcher.

## Ethics Statement

The studies involving human participants were reviewed and approved by the Institutional Review Board (IRB) of Vanderbilt University (160658). The patients/participants provided their written informed consent to participate in this study.

## Author Contributions

MD contributed the data. RC and SP designed the analyses and wrote the manuscript. RC performed statistical analysis. All authors contributed to the research questions, theoretical development, and manuscript revision.

### Conflict of Interest

The authors declare that the research was conducted in the absence of any commercial or financial relationships that could be construed as a potential conflict of interest.
